# Unlocking the potential of tropical root crop biotechnology in east Africa by establishing a genetic transformation platform for local farmer-preferred cassava cultivars

**DOI:** 10.3389/fpls.2013.00526

**Published:** 2013-12-24

**Authors:** Evans Nyaboga, Joshua Njiru, Edward Nguu, Wilhelm Gruissem, Herve Vanderschuren, Leena Tripathi

**Affiliations:** ^1^International Institute of Tropical AgricultureNairobi, Kenya; ^2^Department of Biology, Plant Biotechnology, Eidgenössische Technische HochschuleZurich, Switzerland; ^3^Department of Biochemistry, University of NairobiNairobi, Kenya

**Keywords:** cassava, friable embryogenic callus, *Agrobacterium tumefaciens*, genetic transformation, farmer-preferred cultivars

## Abstract

Cassava genetic transformation capacity is still mostly restricted to advanced laboratories in the USA, Europe and China; and its implementation and maintenance in African laboratories has remained scarce. The impact of transgenic technologies for genetic improvement of cassava will depend largely on the transfer of such capabilities to researchers in Africa, where cassava has an important socioeconomic niche. A major constraint to the development of genetic transformation technologies for cassava improvement has been the lack of an efficient and robust transformation and regeneration system. Despite the success achieved in genetic modification of few cassava cultivars, including the model cultivar 60444, transgenic cassava production remains difficult for farmer-preferred cultivars. In this study, a protocol for cultivar 60444 developed at ETH Zurich was successfully implemented and optimized to establish transformation of farmer-preferred cassava cultivars popular in east Africa. The conditions for production and proliferation of friable embryogenic calli (FEC) and *Agrobacterium-mediated* transformation were optimized for three east African farmer-preferred cultivars (Ebwanatereka, Kibandameno and Serere). Our results demonstrated transformation efficiencies of about 14–22 independent transgenic lines per 100 mg of FEC for farmer-preferred cultivars in comparison to 28 lines per 100 mg of the model cultivar 60444. The presence, integration and expression of the transgenes were confirmed by PCR, Southern blot analysis and histochemical GUS assay. This study reports the establishment of a cassava transformation platform at International Institute of Tropical Agriculture (IITA) hosted by Biosciences eastern and central Africa (BecA) hub in Kenya and provides the basis for transferring important traits such as virus resistance and prolonged shelf-life to farmer-preferred cultivars in east Africa. We anticipate that such platform will also be instrumental to transfer technologies to national agricultural research systems (NARS) in sub-Saharan Africa.

## Introduction

Cassava (*Manihot esculenta* Crantz) is the third most important source of calories in the tropics, after rice and maize (FAOSTAT, 2008). Millions of people depend on cassava in Africa, Asia and Latin America. It is vital for food security as well as income generation for farmers including poor farmers, many of them women, growing cassava on marginal land. Global production of cassava is about 256 Million tonnes, out of which 146 Million tonnes are produced in Africa (FAOSTAT, 2012). The global demand for cassava is rapidly growing because of its increasing use by the starch industry and its good potential for biofuel production (Jansson et al., 2009). Despite its importance there are traits that need improvement, such as pest and disease susceptibilities, accumulation of cyanogens, and post-harvest physiological deterioration (Ceballos et al., 2004; Sayre et al., 2011). Due to the high heterozygosity, allopolyploidy, low fertility as well as unsynchronized flowering of cassava, conventional breeding is difficult and time-consuming (Ceballos et al., 2004). As an alternative, genetic transformation offers great potential for cassava improvement (Liu et al., 2011).

The ability to use biotechnological tools to improve cassava was proved possible in the mid 1990s. Li et al. (1996) reported *Agrobacterium*-mediated transformation of somatic cotyledons to regenerate transgenic shoots by organogenesis. Simultaneously, Schöpke et al. (1996), demonstrated regeneration of transgenic plantlets through microparticle bombardment of embryogenic cell suspensions. In the latter system, transformation relied on the generation of embryogenic cell clusters, known as friable embryogenic callus (FEC) as the target material for transformation. However, some of the disadvantages of microprojectile bombardment are that the transformation efficiency may be lower than with *Agrobacterium*-mediated transformation and the device and consumables are costly. Also *Agrobacterium*-mediated transformation offers several other advantages over microprojectile bombardment procedure, such as the possibility to transfer only one or few copies of DNA fragments carrying the genes of interest at higher efficiencies with lower cost and the transfer of very large DNA fragments with minimal rearrangement (Shibata and Liu, 2000; Gelvin, 2003). Therefore, plant transformation through *Agrobacterium*-mediated DNA transfer has become a favored approach for many crop species (Barampuram and Zhang, 2011). For the generation of transgenic cassava, *Agrobacterium*-mediated transformation of FEC, a combination of the two original systems, has become the most efficient and preferred strategy (Liu et al., 2011).

The FECs are preferred tissue for transformation as it reduces the risk of generating chimeric plants compared to procedures using organized tissues, such as cotyledons (González et al., 1998). Protocols using FEC also appeared suitable for large production of independent transgenic events particularly with the model cultivar 60444 (Bull et al., 2009). However, despite the original techniques being published approximately 18 years ago, the uptake and success rate by different laboratories has been poor, particularly in sub-Saharan Africa, where cassava is an important staple crop. The lack of uptake of the technology in Africa has been largely attributed to the limited number of well equipped laboratories and technique(s) being complicated and labor-intensive, but also the difficulty in adapting the protocols developed for the model cultivar 60444 with farmer-preferred cultivars. Long-term success of transgenic technologies for genetic improvement of cassava will depend largely on the transfer and expansion of such capabilities to researchers in Africa, where these systems can be exploited for specific local needs in the relevant germplasm (Machuka, 2001; Toennissen et al., 2003; Vanderschuren, 2012).

Although a tropical crop, the technical expertise required to develop and apply genetic transformation technology in cassava is still mostly restricted to advanced laboratories; and its implementation and maintenance in laboratories in developing countries has remained scarce. A critical issue for the successful adoption of transgenic cassava by farmers in Africa is establishment of well equipped laboratories and the development of human resource capacity to produce transgenic cassava using farmer-preferred cultivars with important agronomic traits (Ezezika et al., 2012). Hundreds of genetically distinct varieties of the crop are known to exist, however, only a very small fraction of these will ever be targeted within genetic engineering programs. Therefore, there is a need to develop capacities to transform the most important local cultivars and landraces (Taylor et al., 2002). Because transgenic strategies to improve cassava now being evaluated in the field (Zhang et al., 2010; Koehorst-van Putten et al., 2012; Ogwok et al., 2012) are so far restricted to two cultivars, it is also important to assess the technology in cassava genotypes adapted to the respective field environments. Locally adapted cultivars and landraces have often been selected and adopted by farmers because of particular improved traits (Kawuki et al., 2011). Therefore, transgenic programs must generate products readily acceptable to the intended end users.

To date, effective FEC-based transformation protocols have been developed mainly for cultivar 60444, which is considered as a model African cassava cultivar (Bull et al., 2009; Taylor et al., 2012). Although this cultivar could be used for research purposes, it is not anymore cultivated by farmers because of low yield, low nutritional quality and susceptibility to viral and bacterial diseases. Previous studies using the FEC-based cassava transformation method reported the introduction of important agronomic traits, such as resistance to African cassava mosaic virus (ACMV) and cassava brown streak virus (CBSV) in cultivars 60444 and TME7 (Zhang et al., 2005; Vanderschuren et al., 2009, 2012; Yadav et al., 2011). A procedure to transform landraces from the TME series has been recently developed (Zainuddin et al., 2012) at ETH Zurich and also used successfully to transform the industry-preferred cultivar T200 in South Africa (Chetty et al., 2013). However, transformation of cassava cultivars particularly preferred by African farmers remains challenging because FEC production tends to be genotype-dependent and regeneration of transformed FEC is often problematic (Raemakers et al., 2001; Chetty et al., 2013).

Here we report the successful implementation of a robust cassava transformation platform at IITA hosted by BecA hub in Nairobi and its use for the generation of transgenic cassava of local farmer-preferred cultivars selected based on their economic importance, frequency of cultivation in different geographical and climatic zones of east Africa, and susceptibility to major production constraints in sub-Saharan Africa.

## Materials and methods

### Cassava material

**Figure 1:**
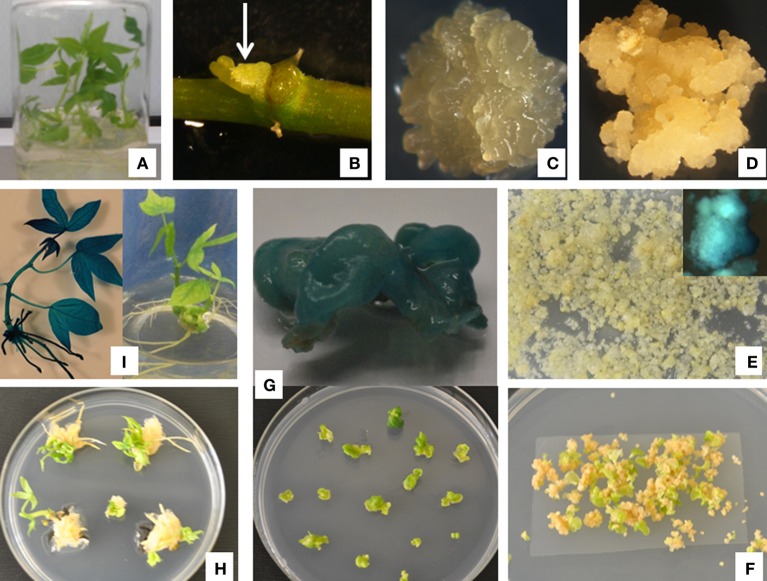
***Agrobacterium*-mediated transformation of cassava cultivar Serere using FEC. (A)** *In vitro* shoot culture; **(B)** axillary bud (arrow); **(C)** primary somatic embryos; **(D)** friable embryogenic callus; **(E)** *Agrobacterium*-inoculated FEC proliferating on selective medium and transient GUS assay (upper right corner); **(F)** somatic embryos/cotyledons on selective embryo development and maturation medium; **(G)** mature somatic embryos on shoot elongation medium and GUS positive somatic embryos (up); **(H)** shoots developing on shoot elongation medium; **(I)** transgenic plantlets germinated on basic cassava medium and GUS expression in shoot (left).

Cassava cultivars (Ebwanatereka and Serere) were obtained from Kenya Agricultural Research Institute (KARI), Kenya and cultivar 60444 was obtained from ETH, Zurich. The cultivars Mkombozi, Kibandameno, Albert, Kibaha and TME14 were obtained from germplasm collection at IITA. All the cultivars were maintained as shoot cultures on cassava basic medium (CBM; Supplementary Table 1) at 28°C under a 16/8 h photoperiod (Figure 1A).

### Somatic embryogenesis

Nodal explants were cut and placed horizontally on cassava axillary bud medium (CAM; Supplementary Table 1) for 3–6 days, depending on the cultivar used. The enlarged axillary buds (AB; Figure 1B) were removed from the nodal explants with a sterile hypodermic needle and transferred onto embryo induction medium (CIM; Supplementary Table 1). For immature leaf lobe (ILL), 2–6 mm length explants were excised from *in vitro* mother plantlets and placed on CIM. Organized embryogenic structures (OES) were induced from axillary buds (AB) and immature leaf lobe (ILL) explants, according to procedure previously described (Bull et al., 2009; Taylor et al., 2012). A hundred and forty explants were used in each experiment. Explants were incubated for 4 weeks at 28°C in the dark and subculturing at 14 days. The comparative potential of somatic embryogenesis was evaluated based on the frequency (*x*/140 explants) of OES production for each cultivar.

### Production and maintenance of friable embryogenic callus (FEC)

Production of FEC was performed according to the protocols described by Bull et al. (2009) with modifications. Primary organized embryogenic clusters were transferred to Greshoff and Doy (GD) medium (Gresshoff and Doy, 1974; Supplementary Table 1) supplemented with 50 μM of picloram and incubated at 28°C in dark. FECs were subsequently grown under 16/8 h photoperiod and 28°C conditions and sub-cultured onto fresh media every 3 weeks. The effect of L-tyrosine on FEC production in cassava cultivars was evaluated by transferring OES onto GD medium supplemented with 50 μM picloram in combination with different concentrations of tyrosine (125, 250, and 500 μM).

### Regeneration of FECs

Assessment of the FEC regeneration potential was performed with 50 mg of FEC tissues spread on mesh and cultured on embryo maturation and germination media (MSN; Supplementary Table 1) at 28°C under 16/8 h photoperiod. The emerging green cotyledons were counted, removed and placed on cassava shoot elongation medium (CEM; Supplementary Table 1), while the remaining FECs were transferred to fresh media every 10 days. The regeneration capacity was measured as the total number of green cotyledons produced over 100 days. Germinating shoots were transferred to cassava basic medium (CBM, Supplementary Table 1) for establishment of plantlets. The regeneration process was monitored as the total number of green cotyledons produced within 100 days and the total number of plants regenerated.

### *Agrobacterium* and binary vectors

*Agrobacterium tumefaciens* strain LBA4404 carrying the binary vector pCAMBIA1301 was used in this study. The pCAMBIA1301 (GenBank AF234297) contains hygromycin phosphotransferase gene (*hpt*) as selection marker and β-glucuronidase (*gusA*) reporter gene with a plant intron, both driven by the constitutive Cauliflower mosaic virus promoter (CAMV 35S) (Supplementary Figure 1). The binary vector was transformed into *Agrobacterium tumefaciens* strain LBA4404 by electroporation. Single colonies from LB agar plates containing kanamycin (50 mg l^−1^), rifampicin (50 mg l^−1^) and streptomycin (100 mg l^−1^) were used to initiate 2 ml LB medium as starter cultures. After 48 h shaking at 150 rpm at 28°C, this suspension was used to inoculate a 20 ml YEP medium containing the same antibiotics, and grown overnight on a shaking platform at 150 rpm to reach an OD_600_ of 0.75–1.0. Bacteria were spun down and washed twice with liquid GD medium. The pellet of bacteria was re-suspended in liquid GD medium supplemented with 200 μM acetosyringone (Sigma Chemical Co.) to an OD_600_ of 0.5.

### Transformation, selection and regeneration of transgenic plants

Three month old FECs (100 mg) were co-cultivated with 10 ml of the *Agrobacterium tumefaciens* strain harboring pCAMBIA1301 in sterile 50 ml falcon tubes, mixed vigorously to disaggregate the callus tissues and incubated for 30 min with gentle shaking (40 rpm). *Agro*-inoculated FEC tissues were transferred onto a 100 μm mesh placed on sterilized paper towel for 5 min to remove excess bacteria. The FEC were co-cultivated on GD medium for 3 days with *Agrobacterium* under light (16/8 h photoperiod) at 22°C. Following the co-cultivation step, *Agro*-inoculated FEC were washed 3 times with liquid GD medium containing 500 mg l^−1^carbenicillin and transferred to mesh. The mesh was placed on GD media supplemented with 250 mg l^−1^carbenicillin and incubated for 4 days of recovery at 28°C 16/8 h photoperiod. After 4 days of incubation, the mesh was transferred to fresh GD medium supplemented with 250 mg l^−1^ carbenicillin and 5 mg l^−1^ hygromycin and kept under 16/8 h photoperiod at 28°C for 7 days. This step was repeated twice with gradually increasing the hygromycin selection to 8 and 15 mg l^−1^. Following the FEC selection on GD media, the mesh with FEC was transferred to MSN medium supplemented with 250 mg l^−1^ carbenicillin and 15 mg l^−1^ hygromycin and kept under 16/8 h photoperiod at 28°C with fortnightly subculturing on fresh MSN medium.

Matured embryos developing cotyledons on selective MSN medium were transferred to CEM supplemented with 100 mg l^−1^ carbenicillin with fortnightly subculturing onto fresh CEM medium for cotyledon development and shoot elongation. The elongated shoots were transferred to CBM for rooting. Rooted plantlets were screened for escapes by transferring stem cuttings to CBM supplemented with carbenicilin 50 mg l^−1^ and hygromycin 10 mg l^−1^ as previously described (Bull et al., 2009). Rooting of the plantlets on selective media was recorded after 2–3 weeks.

The well rooted plantlets were transferred to plastic pots (10 cm diameter) containing sterile coconut peat. Pots were placed in trays and covered with a transparent cover and placed in a glasshouse at 28°C. The cover was opened partially after a week and removed after 2 weeks. The hardened plantlets (10 cm in height) were transferred into bigger plastic pots (30 × 40 cm) for further development of tuberous roots and growth monitoring under glasshouse conditions.

### Histochemical GUS assays

Histochemical GUS assays for transient gene expression was performed 3 days after co-cultivation according to the modified procedure of Jefferson (1987) as described by Bull et al. (2009) for cassava. Transient GUS expression frequencies were determined by expressing GUS positive calli as a proportion (%) of the total number of calli in the sample. Stable GUS expression was checked using callus, cotyledonary stage embryos and hygromycin-resistant regenerated plants.

### Molecular analysis of transgenic lines

Both PCR and Southern blot analyses were carried out to confirm the transformation events. The total genomic DNA was extracted from *in vitro* grown shoots using the cetyltrimethylammonium bromide (CTAB) method (Soni and Murray, 1994). For PCR analysis the *hpt* and *gusA* genes were amplified to confirm the integration of the transgene. The primer sequences were forward primer 5′-AAAGTGTGGGTCAATAATCAGG-3′ and reverse primer 5′-ATGGATTCCGGCATAGTTAAAG-3′ designed to amplify a 215 bp fragment from the *gus*A gene; forward primer 5′-GATGTTGGCGACCTCGT-3′ and reverse primer 5′-GTGTCACGTTGCAAGACCTG-3′ amplifying a 415 bp fragment from the *hpt* gene.

For Southern blot hybridization 15 μg of DNA was digested with 3 U μg^−1^ *Hind*III (New England Biolabs Inc. MA, USA) overnight at 37°C and resolved on 0.8% agarose gels. DNA was blotted onto Hybond-N+ nylon membrane (Roche) and fixed by cross-linking in a STRATA-LINK™ UV cross-linker. The blots were hybridized with digoxigenin (DIG)-labeled *gusA*-specific probe generated using a PCR DIG Probe Synthesis Kit (Roche Applied Sciences, Mannheim, Germany). Hybridization and detection were carried out using a DIG Luminescent Detection Kit for Nucleic Acids (Roche Applied Sciences, Mannheim, Germany), according to the manufacturer's instructions.

### Data analysis

All the experiments were repeated three times and data for all parameters were analyzed by analysis of variance (ANOVA) using PROC ANOVA of SAS 9.2 (SAS Institute, Cary, NC), and Duncan's new multiple range test was used to detect significant differences between means.

## Results and discussion

### Selection of accessions preferred in east Africa

The identification of cassava cultivars that will be readily adopted by significant numbers of farmers in sub-Saharan Africa is essential for the development of genetically modified (GM) product. Resource poor farmers prefer cultivars that offer several important traits like cooking quality, time to maturity, yield and disease resistance. The local cultivars preferred in east Africa (Serere, Ebwanatereka, Kibandameno, Mkombozi, Kibaha, Albert and TME14) were selected, based on consultation with cassava breeders, for establishment of genetic transformation system. These cultivars were selected due to their economic importance and wide cultivation across several countries in east Africa (Table 1). The selected cultivars are high-yielding and have desirable root quality attributes such as taste, mealiness, texture, aroma, flavor, cooking qualities and market value (National Cassava Programme-Uganda Report, 2005). However, all these cultivars are highly susceptible to both CMD and cassava brown streak disease (CBSD) except TME14, which is only susceptible to CBSD (Ntawuruhunga and Legg, 2007). All these cultivars are also highly susceptible to cassava bacterial blight (CBB) and post-harvest physiological deterioration (PPD). Resistance to CMD, CBSD, CBB as well as prolonged shelf life is therefore considered as priority traits for these cultivars. The aforementioned cultivars with improved traits would certainly be perceived favorably and adopted, if available, by small landholder farmers in east Africa.

**Table 1:** **Origin, agronomic traits and tuber quality attributes of selected cassava cultivars and landraces preferred in east Africa**.

**Cassava cultivar**	**Origin**	**Traits and reaction to diseases**	**Cultivated in country**
Serere	CIAT	High dry matter (40%), low cyanogenic potential, high yielding (30 t/ha), white cotex, mature in 8–9 months, erect growth, CMD susceptible, CBSD tolerant	Kenya
Ebwanatereka	Landrace	High dry matter (39%), high yielding (30 t/ha), mealiness, highly CMD and CBSD susceptible, PPD susceptible	Kenya and Uganda
Albert	Landrace	Low cyanogenic potential, yield (15 t/ha), CMD tolerant and CBSD susceptible	Tanzania and Kenya
Kibaha	Landrace	High dry matter, low CNP, yield (20 t/ha), CMD and CBSD susceptible	Tanzania
TME 14	IITA	High dry matter (39%), high yielding (23 t/ha), low cyanogenic potential, sweet, low branching, white cortex, mature in 9 months, CMD tolerant and CBSD susceptible, PPD susceptible	Kenya, Uganda and Tanzania
Kibandameno	Landrace	High dry matter (40%), low cyanogenic potential, sweet, high bulk, high yielding (30 t/ha), maturing 8–12 months, CMD and CBSD susceptible, PPD susceptible	Kenya and Tanzania
Mkombozi	Landrace	High dry matter, low cyanogenic potential, high yielding (25 t/ha), CMD tolerant and CBSD susceptible	Kenya and Tanzania
60444	IITA	Model cultivar	No more in use by farmers

### Production of FEC of farmer-preferred cultivars

Successful genetic transformation of cassava using FECs depends initially on the production of OES, and the ability of OES to induce and proliferate into pure and homogeneous FEC (Taylor et al., 2004; Liu et al., 2011). In this study, high quality OES were obtained from both AB and ILL explants of all cassava cultivars tested. OES induction efficiencies ranged from 65 to 86% for AB explants and 62–85% for ILL explants (Table 2). Depending on the cultivar, the formation of OES was observed on AB and ILL explants after 10–14 and 18–28 days, respectively. The highest frequencies of somatic embryogenesis for cultivars 60444, Ebwanatereka and Kibandameno were obtained using AB explants (Table 2). The cultivar Serere performed equally well using AB and ILL explants producing OES frequencies of 84 and 85%, respectively (Table 2; Figure 1C). The induction of primary embryogenic tissues from the explant is considered to be the most important step during the production of target tissues for gene insertion in cassava. Failure to establish efficient procedures for this step, in order to eventually produce FEC tissues, renders cassava genetic transformation impossible. In this study, we demonstrated, for the first time, OES production in farmer-preferred cultivars of cassava using two different types of explants.

**Table 2:** **Average frequencies of somatic embryos induced from different explants of various cassava cultivars**.

**Cassava cultivar**	OES production frequency (%)^*^	
	**Axillary buds (AB)**	**Immature leaf lobes (ILL)**
60444	86.19 ± 6.07	73.57 ± 11.09
Ebwanatereka	80.95 ± 3.22	74.29 ± 8.24
Serere	84.29 ± 9.37	85.48 ± 2.89
Kibandameno	83.39 ± 8.23	70.24 ± 3.84
Mkombozi	81.79 ± 7.57	64.36 ± 9.37
Kibaha	69.52 ± 10.21	62.86 ± 9.29
Albert	65.71 ± 7.73	70.24 ± 10.53
TME14	78.57 ± 7.25	65.71 ± 8.95

* *Percentage OES produced from 140 explants; OES production frequencies were recorded by calculating the ratio of OES clusters/cultured explants. Data represents means ± SD of three independent experiments*.

**Figure 2:**
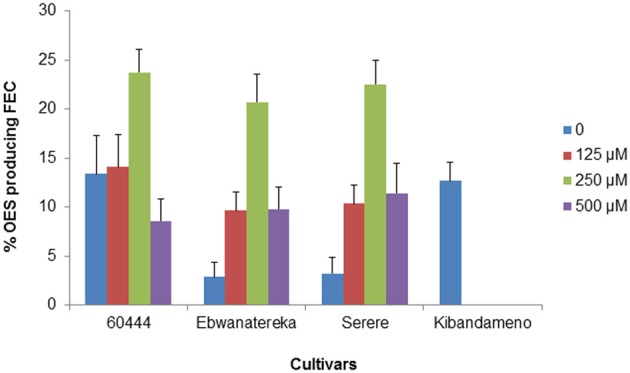
**Effect of L-tyrosine on the production of FEC from different cassava cultivars**. FEC production frequencies were recorded by calculating the ratio of FEC clusters/OES cultured. Values are means ± *SD* of three independent experiments.

The generation and propagation of FEC is fundamental for successful and efficient transformation. Protocols for generation of FEC have been lacking for east African cassava cultivars, thereby delaying their genetic engineering for agronomic and other desirable traits. Work at IITA-Nairobi in collaboration with ETH Zurich led to optimizing conditions suitable for production and regeneration of FEC from the selected cassava cultivars. FEC were obtained for three local cultivars (Ebwanatereka, Serere and Kibandameno) in addition to model cultivar 60444 (Figure 1D), whereas the other four local cultivars (Mkombozi, Albert, Kibaha and TME14) did not produce any FEC. Our results suggests that FEC production is cultivar-dependent and it is possible that Mkombozi, Albert, Kibaha and TME14 need further optimization of tissue culture conditions, media composition and timing to produce FEC. Supplementation of L-tyrosine at 250 μM into FEC induction medium significantly enhanced OES conversion into FEC in three cultivars (Ebwanatereka, Serere and 60444) where as FEC production and proliferation were impeded on L-tyrosine-based media for Kibandameno (Figure 2), suggesting that the L-tyrosine effect is also genotype-dependent. The increase of 1.7–7.2 fold in FEC production was recorded for Ebwanatereka and Serere, respectively, when OES were placed on GD medium supplemented with 250 μM L-tyrosine.

The genotypic effect on FEC production of cassava genotypes tested in this study was also observed in the FEC production of a range of Asian cassava genotypes (Raemakers et al., 2001) and recognized as one of the major limitations of the application of FEC in genetic transformation of farmer-preferred cultivars of cassava (Taylor et al., 1997; Fregene and Puonti-Kaerlas, 2002; Hankoua et al., 2005; Liu et al., 2011). These observations demonstrate that there might be an underlying genetic control in the capability of a given genotype to induce and proliferate FECs. The time required to generate FEC from OES also varied between cultivars, ranging from 9 to 22 weeks. Kibandameno was similar to cultivar 60444 and initiated FEC production within 9–11 weeks. Formation of FEC from cultivar Serere was also relatively rapid (12 weeks), whereas Ebwanatereka took 18–22 weeks to form FEC. Depending on the cultivar, 3–6 subcultures of 21 days each resulted in rapidly proliferating, pale yellow FECs. Such variations in time required to obtain FEC cultures imply that cultures should be screened for sufficient periods of time before any conclusion on the response of a cultivar is made.

Since genetic transformation system depend on regeneration of transformed FEC tissue and each cassava cultivar may not respond in those reported condition, we evaluated the regenerative potential of FEC of the three farmer-preferred cultivars (Serere, Ebwanatereka and Kibandameno) and compared with cultivar 60444. The regeneration potential of FEC from these cultivars is presented in Table 3. All the cultivars tested were highly regenerative, producing an average of over 40 cotyledon-stage embryos per 50 mg of FEC (Table 3). More cotyledon-stage embryos were produced from FECs of Serere in comparison to cultivar 60444. In total 54, 50, 40, and 37 cotyledon-stage embryos were produced from Serere, 60444, Ebwanatereka and Kibanadameno, respectively, over 100 days (Table 3). There was no significant difference (*p* ≤ 0.05) in germination of cotyledon-stage embryos of Serere in comparison to cultivar 60444, whereas germination of cotyledon-stage embryos of Ebwanatereka and Kibandameno was significantly (*p* ≤ 0.05) lower. The differences in regeneration efficiencies would be due to genotypic differences. The relatively high germination frequencies of FECs from Serere, Ebwanatereka, Kibandameno and cultivar 60444 suggested that FEC tissues produced from these cultivars could be suitable for genetic transformation. It was, however, noted that not all somatic embryos produced plantlets showing poor correlation between capacity to form somatic embryos and plant regeneration.

**Table 3:** **Regeneration of complete plants from FECs of four cultivars of cassava**.

**Cassava cultivar**	**Cotyledonary-stage embryos recovered per 50 mg FEC**	**Average germination of cotyledonary-stage embryos (%)**	**Plants regenerated per 50 mg FEC**
60444	50.75 ± 5.90^b^	52.17 ± 4.55^c^	17.75 ± 5.56
Serere	54.75 ± 3.80^c^	54.13± 6.44^c^	24.5 ± 5.26
Ebwanatereka	40 ± 11.81^a^	47.29 ± 3.88^b^	16.5 ±6.44
Kibandameno	37 ± 8.44^a^	44.32 ± 5.64^a^	14.5 ± 4.48

*Values are means ± SD of three independent experiments. Values in a column followed by different letters are significantly different from each other at p ≤ 0.05*.

### Establishment of transformation method for farmer-preferred cultivars

In this study, we demonstrated successful and reproducible transformation and regeneration system for three local farmer-preferred cassava cultivars (Serere, Ebwanateraka and Kibandameno) commonly grown in east Africa, using the modified protocol of Bull et al. (2009). Transformation and regeneration of cultivar 60444 required minimal trouble shooting, provided that the experimental parameters were kept as close as possible to those stipulated in the protocol described by Bull et al. (2009) whereas modifications of the protocol such as supplementation of L-tyrosine were required to generate FEC and establish the transformation of some of the local farmer-preferred cultivars. Central to success with this system is the production of sufficient quantity of quality FECs as target tissues for genetic transformation.

Previously, *Agro*-inoculation was done by dropping *Agrobacterium* liquid suspension directly to FEC clusters on the propagation solid medium (Bull et al., 2009). However, in this study, *Agro*-inoculation was modified by immersing FEC in *Agrobacterium* cell suspension for 30 min at room temperature with gentle shaking at 40 rpm. This allowed efficient *Agrobacterium* contact on FEC cells for subsequent infection process as reported in other crops such as banana (Tripathi et al., 2012). Following co-inoculation with *Agrobacterium* transformed FECs proliferated into small clusters of pale yellow colored calli on hygromycin selection media (Figure 1E). Clusters of transformed FECs started developing into somatic embryos after transfer to MSN media (Figure 1F). After seven cycles of 10 days each on MSN, about 140–240 putatively transformed somatic embryos were produced depending upon cultivars (Table 4). Somatic embryos from the aforementioned cultivars maturing on selective MSN media were transferred to elongation media (CEM), where about 26–45% somatic embryos germinated (Figures 1G,H; Table 4). Serere performed significantly (*p* ≤ 0.05) better in comparison to model cultivar 60444 at this stage of regeneration of transformants, whereas Ebwanatereka and Kibandameno had a significantly (*p* ≤ 0.05) lower efficiency of somatic embryo germination (Table 4). Germination of somatic embryos is accompanied by the activation in expression of myriad of genes encoding photosynthetic and chloroplast components (Baba et al., 2008). Time differences in activation of these genes might have caused variation in germination of somatic embryos derived from the different local cassava cultivars. Transgenic lines were regenerated in a 3–5 month period after *Agro*-inoculation of FECs of cultivar 60444, Serere, Ebwanatereka and Kibandameno (Table 4; Figure 1I). Previous studies together with our findings suggest that, the regeneration of plants from cassava somatic embryos is a challenging task, especially due to the low frequency of embryo germination, and this caveat has prevented the generation of transgenic cassava from a wider range of genotypes. Transformation frequency of the model cultivar 60444 was slightly higher than Serere, Ebwanatereka and Kibandameno. We obtained about 22, 17, and 14 transgenic lines from 100 mg FEC (approximately 20 FEC clusters) of Serere, Ebwanatereka and Kibandameno, respectively, whereas about 28 transgenic lines were produced from 100 mg of FEC of cultivar 60444. However, Bull et al. (2009) reported production of 50 transgenic lines from 100 FEC clusters of cultivar 60444 and slightly lower efficiencies for TME accessions were reported (Zainuddin et al., 2012). Chetty et al. (2013) reported 45 transgenic lines from 140 FEC clusters of cultivar T200. Taylor et al. (2012) obtained 14–28 transgenic lines per cm^3^ of settled cell volume of FECs of 60444. Comparing transformation efficiencies in cassava is complicated because of differences in genotypes, tissue culture system, transformation methods and even differences in regeneration capacity among FEC lines derived from the same genotype. Therefore, variability of transformation efficiencies between independent procedures cannot be eliminated. This study has shown successful transformation system for cultivars Serere, Ebwanatereka and Kibandameno, for which transformation had not previously been reported. Although, Taylor et al. (2012) reported a platform for production of transgenic cassava, but it was only reported for the model cultivar 60444 and not tested for any of the farmer-preferred cultivars.

**Table 4:** Regeneration and validation of transgenic plants of various cultivars of cassava using FECs^*^.

**Cassava cultivar**	**Percentage cell cluster showing transient GUS expression**	**No. of somatic embryos on selection**	**Germinated embryos (%)**	**Regenerated plants^ȼ^**	**PCR positive (%)**
60444	61.38	202^c^	36.6^c^	28 (37.8%)^c^	100
Serere	53.51	240^d^	45.0^d^	22 (20.4%)^b^	100
Ebwanatereka	46.15	187^b^	25.7^a^	17 (35.4%)^a^	100
Kibandameno	51.20	141^a^	30.5^b^	14 (32.6%)^a^	100

* 100 mg of FEC for each cultivar was used in each experiment;

ȼ Percentage with respect to germinated embryos. Values in a column followed by different letters are significantly different from each other at p ≤ 0.05.

**Figure 3:**
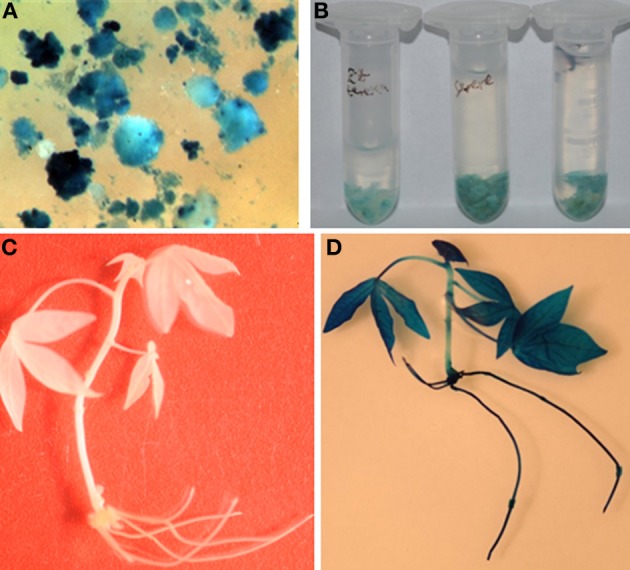
**Transient and stable expression of GUS in co-cultivated calli and hygromycin-resistant transformants. (A)** transient GUS expression after 3 days of co-cultivation; **(B)** stable GUS expression in somatic embryos of Ebwanatereka (left), Serere (middle) and cultivar 60444 (right); **(C)** no GUS expression in non-transgenic control plant; **(D)** stable expression of the GUS gene in transgenic Serere plantlet.

The most commonly used histochemical analysis in plant genetic engineering programmes is the *gus*A visual marker gene (Jefferson, 1987). This is an invaluable tool for developing genetic engineering technologies in new cultivars and as a tool for teaching and training in the basic aspects of this technology. In this study, transient GUS expression assay after 3 days of co-cultivation showed blue coloration confirming transient expression of the reporter gene in FECs of all the four cultivars (Figures 3A,B). However, variations in the frequency of blue foci were observed among the four cultivars tested (Table 3). The percentage of GUS positive calli ranged from 46 to 61% (Table 4). Cultivar 60444 yielded higher frequencies of transient GUS expression. The hygromycin-resistant calli and mature somatic embryos stained a uniform blue color (Figures 1E and G). GUS assay confirmed the transgenic nature of regenerated plantlets (Figure 1I). A uniform blue coloration was observed in regenerated transgenic plants, confirming stable expression of *gusA* gene throughout the plant whereas no blue coloration was observed in non-transgenic plants (Figures 3C,D).

**Figure 4:**
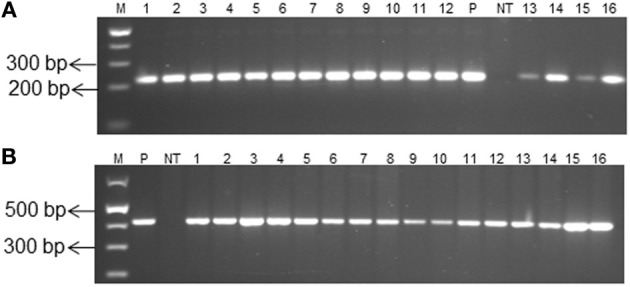
**PCR analysis of transgenic cassava lines using (A) *gus*A (B) *hpt*-specific primers**. Lanes: M, molecular size marker (1 kb plus DNA ladder); NT, non-transgenic plantlet DNA; P, pCAMBIA1301 plasmid DNA as a positive control; 1–8, 9–12, and 13–16, transformed cassava lines of cultivars 60444, Serere and Ebwanatereka, respectively.

A 215 bp amplicon corresponding to the internal fragment of *gus*A gene was amplified from genomic DNA of all selected transgenic plants using *gus*A gene-specific primers (Figure 4A), confirming the presence of *gus*A transgene in transgenic plants. A 415 bp amplicon was observed in all selected transgenic plants using *hpt*-specific primers (Figure 4B), confirming the presence of both gusA and *hpt* genes. Southern blot analysis was performed with *Hind*III-digested genomic DNA in order to confirm the transgene integration and determine the number of inserted copies. Bands corresponding to transgene integration were observed for the selected transgenic plants confirming gene integration in plant genome, whereas no band was observed for the non-transgenic control (Supplementary Figure 2). Several transgenic plants were acclimatized in coconut pit in plastic pots and transferred to soil in pots in the glasshouse. The plants exhibited identical morphology compared with control non-transgenic plants.

### Genetic engineering of target traits

Efforts to improve cassava through genetic engineering have concentrated on a few major traits to complement conventional breeding. The major ones in Africa are resistance to viral and bacterial diseases and tolerance to PPD. Viral diseases are the most important biotic limitation to cassava production in sub-Saharan Africa, and particularly in east Africa, where CBSD and CMD combine to impact the crop (Legg et al., 2011). The development of virus-resistant locally adapted cultivars is restricted by limitations inherent to traditional breeding (Ceballos et al., 2004). In the last two decades, RNAi-based approaches were tested in transgenic cassava and proved to confer robust CMD resistance in the model cassava cultivar 60444 (Vanderschuren et al., 2007, 2009). However, stability of the engineered CMD resistance remains to be demonstrated under field conditions and over multiple cycles of propagation (Donald Danforth Plant Science Center, 2006).

CBSD has recently emerged as an important threat to cassava production in east Africa (Alicai et al., 2007). Because farmer-preferred landraces and newly-bred cassava cultivars deployed in east Africa lack CBSD resistance, genetic engineering has been considered as a promising approach to rapidly implement CBSD resistance in cultivars currently preferred by farmers in east Africa. Laboratories in USA and Europe have demonstrated that CBSD resistance can be engineered in cassava using an RNAi-based approach targeting the coat protein sequence of CBSVs (Yadav et al., 2011; Ogwok et al., 2012; Vanderschuren et al., 2012). These results showed that RNAi approach is a promising technology for engineering CBSD resistance in east African farmer-preferred landraces to reduce the increasing impact of cassava viral diseases.

Cassava production can also be severely affected by cassava bacterial blight (CBB), caused by gram-negative bacteria *Xanthomonas axonopodis* pv. manihotis *(Xam)*. This disease is present in all regions where cassava is grown and production losses can reach up to 80 or 100% (Verdier et al., 2004; Ogunjobi and Dixon, 2006). While there are a few cassava cultivars that show relatively high levels of natural resistance to CBB (Restrepo et al., 2004), they are either not well adapted to particular agroecological regions where cassava is cultivated or do not exhibit the farmer- and/or consumer-preferred characteristics. To our knowledge, transgenic methods to improve resistance to CBB have not been reported yet. Therefore, the knowledge of plant immunity on model plants and other crops, such as *Arabidopsis* and rice, could be brought to cassava. The introduction of PRR genes, such as the *Xa21* from rice or *EFR* (*Elongation Factor Receptor*) in cassava, could provide a broad and durable resistance to CBB, as has been demonstrated for other plants (Lacombe et al., 2010; Mendes et al., 2010). Genetically engineered resistance against bacteria has also been achieved in banana by using genes regulating programmed cell death such as hypersensitive response-assisting protein (*Hrap*) and plant ferredoxin-like protein (*Pflp*) from sweet pepper (*Capsicum annuum*) (Tripathi et al., 2010; Namukwaya et al., 2012). Such an approach could also be used to enhance resistance of cassava against bacterial blight in the near future.

Although cassava yield is high, and its storage root is rich in starch, the PPD of cassava roots seriously affects its storage and utilization (Wheatley et al., 1985). Development of cassava varieties with improved storage performance would benefit farmers, consumers and industries. The total benefits for cassava varieties including delayed PPD were estimated to be in the range of $280 million for Uganda only (Rudi et al., 2010). As a unique biological phenomenon, PPD in cassava is a physiological and biochemical decay process caused by an oxidative burst in storage root cells. PPD has a close relationship with reactive oxygen species (ROS) (Reilly et al., 2004). With the increased study of the temporal and spatial expressions of genes related to ROS production and scavenging in the process of cassava PPD (Reilly et al., 2007; Owiti et al., 2011), as well as the functional verification of these key genes, it is possible to interfere with the PPD process through the regulation of ROS-scavenging activities. The alteration of key enzymes or factors in the PPD pathway might effectively reduce the occurrence of PPD in farmer-preferred cultivars as recently demonstrated in the model cultivar 60444 (Zidenga et al., 2012; Xu et al., 2013).

## Conclusion

Successful application of transgenic technologies in cassava will depend not only on technical advances, but also on successful transfer of knowledge, tools and expertise to the countries in which cassava has an important socioeconomic role. The establishment of cassava transformation platform(s) in institutions in Africa will promote research capabilities and allow scientists autonomy to adapt cassava to suit local agro-ecosystems, ultimately serving to develop a sustainable biotechnology infrastructure in African countries.

To our knowledge this is the first report on successful *Agrobacterium*-mediated transformation of African farmer-preferred cassava cultivars in a laboratory based in sub-Saharan Africa. It is a result of collaborative process involving IITA, Nairobi and ETH, Zurich. The scientific approach followed in this report will serve as model to implement genetic transformation of other cassava cultivars that are agronomically important in Africa. In sub-Saharan Africa, capacities for the production of transgenic crop plants have only recently been developed and remain limited to few crops like banana and maize (Tripathi et al., 2010, 2012; Namukwaya et al., 2012; Ombori et al., 2013). We consider that the present report represents an important step toward building a capacity for transgenic technologies in sub-Saharan Africa. This transformation platform will also acts as an important beginning for the use of transgenic technologies to address cassava production constraints in Africa such as viral and bacterial disease resistance, prolonged shelf life and nutritional enhancement.

In conclusion, a platform for the high throughput production of transgenic cassava has been developed in farmer-preferred cultivars in east Africa at IITA which will serve as a potential platform for training NARS in sub-Saharan Africa for developing improved cassava varieties with important traits like disease resistance.

### Conflict of interest statement

The authors declare that the research was conducted in the absence of any commercial or financial relationships that could be construed as a potential conflict of interest.

## Supplementary material

Table S1 **Composition of media used in tissue culture and transformation experiments**.

Figure S1 **Schematic representation of gene construct used for cassava transformation**. The T-DNA region of construct pCAMBIA1301 with *gus*A and *hpt* genes. LB/RB, left/right T-DNA border sequences; CAMV35SP, CaMV 35S promoter; *hpt*, coding region of the hygromycin B resistance gene; *npt*II, coding region of the neomycin resistance gene; NOS, nopaline synthase; intron-*gus*A, β-glucuronidase containing intron. Arrows indicate direction of transcription.

Figure S2 **Southern blot analyses of genomic DNA of transgenic lines restricted with *Hind*III using PCR-amplified product of *gusA* gene as a probe**. M, DIG-labeled DNA marker; NT, non-transgenic plant; lanes 5, 20 and 22 are transgenic lines of cultivar 60444; 9, 10, and 12 are transgenic lines of cultivar Serere; 18 and 19 are, transgenic lines of cultivar Ebwanatereka.
