# Supplementary material for: Unlocking the potential of tropical root crop biotechnology in east Africa by establishing a genetic transformation platform for local farmer-preferred cassava cultivars
Source: Front Plant Sci. 2013 Dec 24;4:526. doi: 10.3389/fpls.2013.00526 (PMC3872047; doi:10.3389/fpls.2013.00526)
Supplement: Table S1 — Composition of media used in tissue culture and transformation experiments. [file DataSheet1.ZIP › 72190_Tripathi_Supplementary Table 1.pdf]

Supplementary Table 1: Composition of media used in tissue culture and transformation experiments

Name of medium	Composition
Basic shoot culture medium (CBM)	1 × MS[a] salts with vitamins, 2 µM CuSO4, 2% sucrose, 0.3% Gelrite, pH 5.8
Axillary bud enlargement medium (CAM)	1 × MS salts with vitamins, 2 µM CuSO4, 50 µM BAP (6-benzylamino purine), 2% sucrose, 0.8% Noble agar, pH 5.8
Somatic embryo induction medium (CIM)	1 × MS salts with vitamins, 2 µM CuSO4, 50 µM picloram, 2% sucrose, 0.8% Noble agar, pH 5.8
Friable embryogenic calli medium (GD)	1 × GD[b] salts with vitamins, 50 µM picloram, 2% sucrose, 0.8% Noble agar, pH 5.8
Somatic embryo emerging medium (MSN)	1 × MS salts with vitamins, 5 µM NAA (Naphthaleneacetic acid), 2% sucrose, 0.8% Noble agar, pH 5.8
Shoot elongation medium (CEM)	1 × MS salts with vitamins, 2 µM CuSO4, 2 µM BAP, 2% sucrose, 0.8% Noble agar, pH 5.8
Yeast extract peptone broth (YEP)	Yeast extract (0.1%, w/v), beef extract (0.5% w/v), peptone (0.5% w/v), sucrose (0.5% w/v), MgSO4 (0.04% w/v), pH 7.2

[a]MS, Murashige and Skoog (1962); [b]GD, Gresshoff and Doy (1974)
