# Supplementary figures and images for: Unlocking the potential of tropical root crop biotechnology in east Africa by establishing a genetic transformation platform for local farmer-preferred cassava cultivars
Source: Front Plant Sci. 2013 Dec 24;4:526. doi: 10.3389/fpls.2013.00526 (PMC3872047; doi:10.3389/fpls.2013.00526)

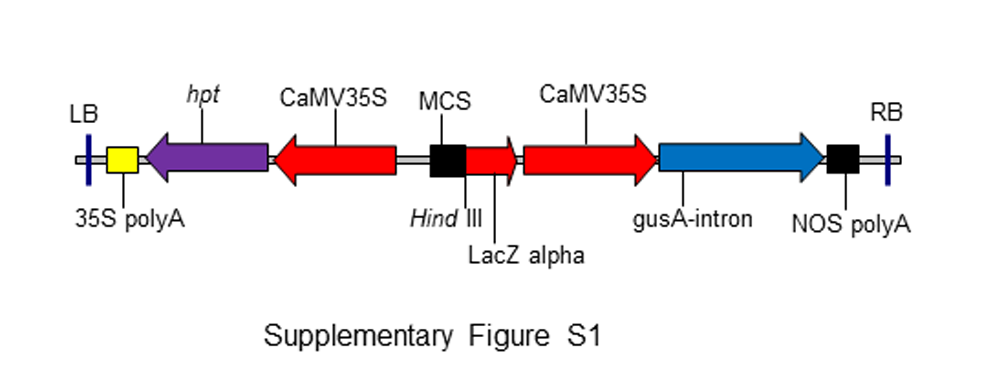

Supplement: Table S1 — Composition of media used in tissue culture and transformation experiments. [file DataSheet1.ZIP › 72190_Tripathi_Figure_5.TIF]

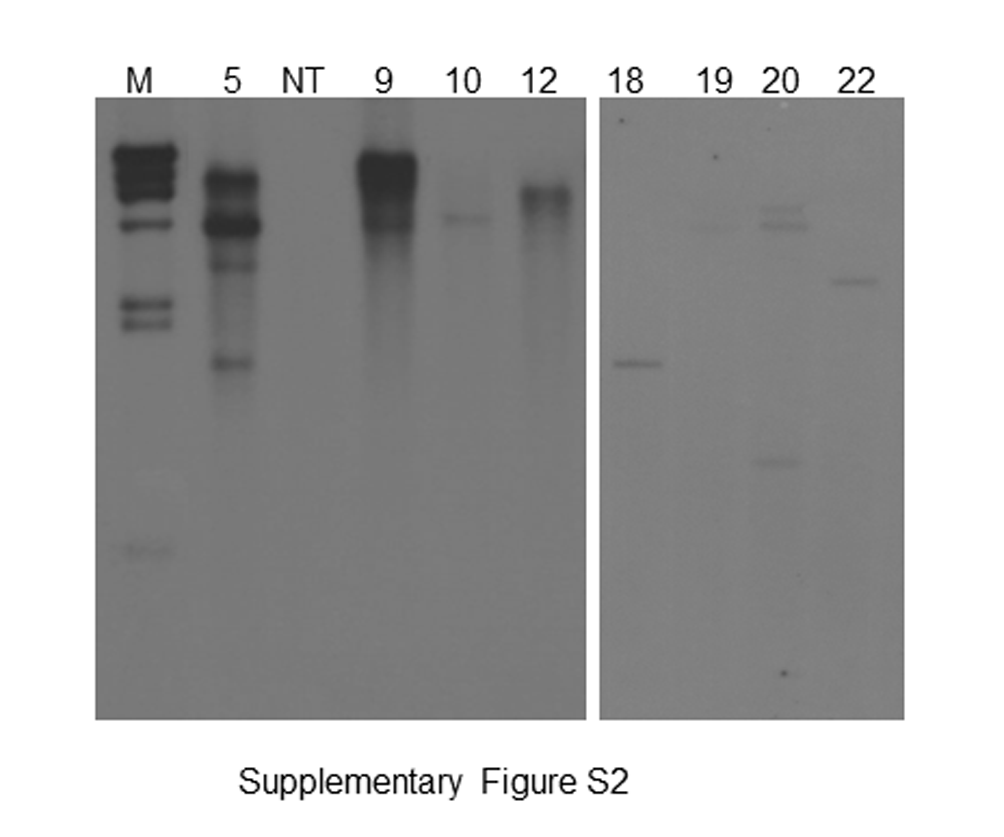

Supplement: Table S1 — Composition of media used in tissue culture and transformation experiments. [file DataSheet1.ZIP › 72190_Tripathi_Figure_6.TIF]
